# LESSONS LEARNED FROM QUALITY IMPROVEMENT DATA COLLECTION FROM ASSISTED LIVING PROVIDERS

**DOI:** 10.1093/geroni/igz038.2161

**Published:** 2019-11-08

**Authors:** Lindsay Schwartz

**Affiliations:** American Health Care Association/National Center for Assisted Living, Washington, District of Columbia, United States

## Abstract

In 2012, the American Health Care Association/National Center for Assisted Living (AHCA/NCAL) launched the Quality Initiative, which was focused on four measurable goals: reduce hospital readmissions; reduce off-label use of antipsychotics; improve staff stability; and improve customer satisfaction. With this initiative, assisted living providers required measures to track their progress. In 2015, to enable benchmarking and comprehensive tracking, these measures were added to LTC Trend Tracker, AHCA/NCAL’s online data collection portal. There have been challenges to getting providers to voluntarily submit data, including time, concerns about privacy, understanding the measures, and learning to use LTC Trend Tracker. We have worked consistently with providers to understand challenges of data submission and assist with lessening the burden such as enabling vendors to upload data. To increase participation, we have learned that measures should be easily understood; measures should be specific and important to providers and consumers; and education and training are important.

